# Single mutations in BraRS confer high resistance against nisin A in *Staphylococcus aureus*

**DOI:** 10.1002/mbo3.791

**Published:** 2019-01-17

**Authors:** Kaoru Arii, Miki Kawada‐Matsuo, Yuichi Oogai, Kazuyuki Noguchi, Hitoshi Komatsuzawa

**Affiliations:** ^1^ Department of Oral Microbiology Kagoshima University Graduate School of Medical and Dental Sciences Kagoshima Japan; ^2^ Department of Periodontology Kagoshima University Graduate School of Medical and Dental Sciences Kagoshima Japan

**Keywords:** ABC transporter, mutation, nisin A, resistance, two‐component system

## Abstract

Nisin A is a lantibiotic produced by *Lactococcus lactis *that is widely used as a food preservative. In *Staphylococcus aureus*, the BraRS two‐component system (TCS) senses nisin A and regulates the expression of the ABC transporter VraDE, which is responsible for nisin A resistance. In this study, we exposed *S. aureus *to a sub‐minimum inhibition concentration of nisin A and obtained three spontaneous mutants that were highly resistant to this lantibiotic, designated as SAN (*S. aureus* nisin resistant) 1, SAN8, and SAN87. In the wild‐type *S. aureus *strain, VraDE expression was induced by nisin A. In contrast, SAN8 and SAN87 showed constitutively high VraDE expression, even in the absence of nisin A, while SAN1 showed higher BraRS expression, which resulted in high VraDE expression in the presence of nisin A. We identified a single mutation in the promoter region of *braXRS *in SAN1, whereas SAN8 and SAN87 had single mutations in *braR* and *braS*, respectively. Interestingly, even the unphosphorylated form of the mutant BraR protein induced VraDE expression. These results indicate that conformational changes in BraS or BraR resulting from the point mutations may result in the constitutive expression of VraDE, allowing *S. aureus* to adapt to high concentrations of nisin A.

## Graphical abstract

In this study, we obtained three spontaneous mutants that were highly resistant to nisin A, designated as SAN (*Staphylococcus aureus* nisin resistant) 1, SAN8, and SAN87. We identified that SAN1 had a single mutation in the promoter region of *braRS* which is a two‐component system, while SAN8 and SAN87 had single mutations in *braR* and *braS*, respectively. Compared to the wild type, these mutations caused to high expression of VraDE which is responsible for nisin A resistance.

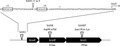

## 1 INTRODUCTION

Many bacteria produce antimicrobial peptides to interfere with other bacteria and promote their own survival within bacterial communities (Cotter, Hil, & Ross, 2005; Jack, Tagg, & Ray, 1995; Nissen‐Meyer & Nes, 1997). Antimicrobial peptides produced by bacteria are called bacteriocins, which in gram‐positive bacteria are generally classified into two groups, class I and class II bacteriocins (Nagao et al., 2006). Class I bacteriocins are also called lantibiotics and contain an unusual amino acid, lanthionine (Nagao et al., 2006), while class II bacteriocins are composed of unmodified amino acids (Nes & Holo, 2000). Lantibiotics are classified into A (linear peptide) and B (globular peptide) types (Nagao et al., 2006; Nes & Holo, 2000). Type A lantibiotics are further classified into two subtypes, type A(I), which includes nisin, subtilin, and epidermin, and type A(II), which includes lacticin 481 and nukacin ISK‐1 (Bierbaum & Sahl, 2009). Many studies have investigated lantibiotics for their clinical use and as food additives (Breukink & de Kruijff, 2006; Field, Cotter, Ross, & Hill, 2015; Gharsallaoui, Oulahal, Joly, & Degraeve, 2016; Shin et al., 2016). The lantibiotic nisin A is produced by *Lactococcus lactis *and is widely used as a food additive throughout the world (Gharsallaoui et al., 2016; Shin et al., 2016). The primary mode of action of nisin A involves its binding to lipid II to inhibit cell wall biosynthesis and promote pore formation in bacterial membranes (Bierbaum & Sahl, 2009).

*Staphylococcus aureus* is a major human pathogen (Foster, 2004; Lowy, 1998; Manders, 1998), causing suppurative diseases, pneumonia, toxic shock syndrome, and food poisoning. Furthermore, *S. aureus* can cause serious problems in patients receiving chemotherapy due to its resistance to many antibacterial agents (Deurenberg et al., 2007; Grundmann, Aires‐de‐Sousa, Boyce, & Tiemersma, 2006; Martens & Demain, 2017). Methicillin‐resistant *S. aureus *(MRSA) is particularly problematic, often causing problems in hospitals and communities (Deurenberg et al., 2007; Grundmann et al., 2006). In genomic studies, *S. aureus* has been observed to possess multiple exogenously acquired genes from transposons, phages, and plasmids that often include antibiotic resistance genes (Bal et al., 2016; Lindsay, 2010).

We and other researchers previously reported on the association between the BraRS two‐component system (TCS) and nisin A resistance (Hiron, Falord, Valle, Débarbouillé, & Msadek, 2011; Kawada‐Matsuo, Yoshida, et al., 2013). In addition, BraDE has also been shown to be involved in nisin A sensing and signaling through BraRS (Hiron et al., 2011). Finally, the phosphorylated BraR protein induces the expression of the ABC transporter VraDE, an intrinsic factor for nisin A resistance. BraRS is also associated with resistance to bacitracin and nukacin ISK‐1, which act upon undecaprenol pyrophosphate and lipid II, respectively (Bierbaum & Sahl, 2009; Islam, et al., 2012). However, because *S. aureus* MW2 showed a relatively low minimum inhibition concentration (MIC) for nisin A (MIC: 512 µg/ml), high concentrations of this lantibiotic have antibacterial activity against *S. aureus* (Hiron et al., 2011; Kawada‐Matsuo, Yoshida, et al., 2013). Many studies have investigated lantibiotics such as nisin A for their clinical use and as food additives (Breukink & de Kruijff, 2006; Field, Cotter, Hill, & Ross, 2015; Gharsallaoui et al., 2016; Shin et al., 2016). To determine whether the application of nisin A could select for a mutant with decreased susceptibility to nisin A, we attempted to isolate such mutants by exposing *S. aureus* cells to a sub‐MIC of nisin A. As a result, we obtained several strains exhibiting a decreased susceptibility to nisin A. We also identified several point mutations in the *braXRS* region resulting in high VraDE expression. These results indicate that endogenous mutations conferring high levels of nisin A resistance in *S. aureus *can arise through exposure of cells to a sub‐MIC of nisin A.

## 2 EXPERIMENTAL PROCEDURES

### 2.1 Bacterial strains and growth conditions

The bacterial strains used in this study are listed in Table 1. *S*. *aureus* and *Escherichia coli *XL‐II were grown in Trypticase soy broth (TSB; Becton Dickinson Microbiology Systems, Cockeysville, MD, USA) and Luria Bertani (LB) broth, respectively. Tetracycline (5 µg/ml) and chloramphenicol (5 µg/ml) were used to select for *S. aureus*, and ampicillin (100 µg/ml) was used to select for *E. coli* when necessary.

**Table 1:** Strains used in this study

Strains	Genotype	Reference
MW2	*Staphylococcus aureus* clinical strain, methicillin‐resistant (*mecA*+)	Grundmann et al. (2006)
MM2070	*braRS* inactivation in MW2, TC^r^	Kawada‐Matsuo, Yoshida, et al. (2013)
MM2156	*braS* inactivation in MW2, TC^r^	Yoshida et al. (2011)
MM2005	*vraDE* inactivation in MW2, TC^r^	Yoshida et al. (2011)
MM2139	*braRS* (MW2) complementation in MM2070, TC^r^, CP^r^	This study
MM2141	*braRS* (SAN8) complementation in MM2070, TC^r^, CP^r^	This study
MM2194	*braR *(MW2) complementation in MM2070, TC^r^, CP^r^	This study
MM2195	*braR *(SAN8) complementation in MM2070, TC^r^, CP^r^	This study
SAN1	nisin A‐resistant mutant from MW2	This study
MM2117	*braRS* inactivation in SAN1, TC^r^	This study
MM2255	*braXRS* (SAN1) complementation in MM2117, TC^r^, CP^r^	This study
SAN8	nisin A‐resistant mutant from MW2	This study
MM2120	*braRS* inactivation in SAN8, TC^r^	This study
MM2145	*braRS* (MW2) complementation in MM2120, TC^r^, CP^r^	This study
MM2147	*braRS* (SAN8) complementation in MM2120, TC^r^, CP^r^	This study
MM2196	*braR *(MW2) complementation in MM2120, TC^r^, CP^r^	This study
MM2197	*braR *(SAN8) complementation in MM2120, TC^r^, CP^r^	This study
MM2116	*braS* inactivation in SAN8, TC^r^	This study
SAN87	nisin A‐resistant mutant from MW2	This study
MM2256	*braRS* inactivation in SAN87, TC^r^	This study
MM2258	*braRS* (SAN87) complementation in MM2120, TC^r^, CP^r^	This study
MM2228	*lacZ* gene fused with the promoter(MW2) of *braRS* in MW2, CP^r^	This study
MM2229	*lacZ* gene fused with the promoter(SAN1) of *braRS* in MW2, CP^r^	This study
MM2264	*lacZ* gene fused with the promoter(MW2) of *braRS *in MM2156, TC^r^, CP^r^	This study
MM2265	*lacZ* gene fused with the promoter(MW2) of *braRS* in MM2116, TC^r^, CP^r^	This study
MM2199	*lacZ* gene fused with the promoter(MW2) of *vraDE* in MW2, CP^r^	This study
MM2200	*lacZ* gene fused with the promoter(MW2) of *vraDE* in SAN1, CP^r^	This study
MM2201	*lacZ* gene fused with the promoter(MW2) of *vraDE* in SAN8, CP^r^	This study
MM2242	*lacZ* gene fused with the promoter(MW2) of *vraDE *in SAN87, CP^r^	This study
MM2262	*lacZ* gene fused with the promoter(MW2‐binding region less) of *vraDE* in MW2, CP^r^	This study
MM2263	*lacZ* gene fused with the promoter(MW2‐binding region less) of *vraDE* in SAN8, CP^r^	This study
MM2266	*lacZ* gene fused with the promoter(MW2‐binding region less) of *vraDE *in SAN87, CP^r^	This study
RN4220	Restriction‐deficient transformation recipient	Kreiswirth et al. (1983)
MM2186	*braR* (MW2) in RN4220, CP^r^	This study
MM2187	*braR* (SAN8) in RN4220, CP^r^	This study
XL‐II	*endA1 supE44 thi‐1 hsdR17 recA1 gyrA96 relA1 lac *[F*´ proAB lacI*q*ZΔM15 *Tn*10 *(TC^r^) Amy CP^r^]	Stratagene
MM1113	His‐tag fused *braD* (MW2) gene in XL‐II, Amp^r^	This study
MM1114	His‐tag fused *braD* (SAN8) gene in XL‐II, Amp^r^	This study

Note

Amp: ampicillin; CP: chloramphenicol; TC: tetracycline.

### 2.2 MIC determination

Minimum inhibition concentrations were determined using a previously described microdilution method (Kawada‐Matsuo, Yoshida, et al., 2013) for nisin A (Sigma‐Aldrich, St. Louis, MO, USA), gallidermin (Santa Cruz Biotechnology, TX, USA), and bacitracin (WAKO Chemicals, Osaka, Japan).

### 2.3 Isolation of spontaneous mutants by nisin A exposure

The *S. aureus* strain MW2 was used to isolate spontaneous mutants that were highly resistant to nisin A using a microdilution method. The MW2 strain was cultured overnight, and an aliquot (containing 10^5^ cells) was inoculated into 100 µl of TSB containing various concentrations of nisin A (Sigma‐Aldrich; twofold dilutions: 16,384 to 16 µg/ml) and incubated at 37°C overnight. Next, using the bacterial cells that grew in the 1/2 MIC of nisin A, the same experiment was repeated two additional times. After the last subculture, the bacterial cells grown in the 1/2 MIC of nisin A were appropriately diluted and plated on TSA. After an overnight incubation, seven colonies were randomly picked and replated on TSA. Subsequently, the MICs of nisin A were determined for the seven strains. This experiment was performed three times independently (experiments 1, 2, and 3).

The expression of VraD (MW2620) was investigated in the strains exhibiting increased MICs for nisin A compared to the wild‐type strain. The *S. aureus* strains were cultured overnight, and an aliquot (containing 10^8^ cells) was inoculated into 5 ml of fresh TSB and grown at 37°C with shaking. When the optical density at 660 nm reached 0.5, nisin A (64 µg/ml) was added to the bacterial culture. After a 15 min of incubation, the bacterial cells were collected and total RNA was extracted using a FastRNA Pro Blue Kit (MP Biomedicals, Solon, OH, USA) according to the manufacturer's protocol. Next, 1 µg of total RNA was reverse‐transcribed to cDNA using a first‐strand cDNA synthesis kit (Roche, Tokyo, Japan). Using the cDNA as template, quantitative PCR was performed using a LightCycler system (Roche, Tokyo, Japan). Primers were designed to amplify MW2620 (*vraD*), and *gyrB* was used as an internal control. The primers used in this assay are listed in Table 2. Finally, the strains exhibiting increased MICs and an increased expression of MW2620 in the absence of nisin A were selected for further analysis.

**Table 2:** Primers used in this study

Target gene ID	Primer—forward	Primer—reverse
Construction of gene‐inactivated mutants		
braR	5′‐ttaggatccaaaatattaattgttgaagatg‐3′	5′‐acaaagcttcttcattttgaaataataacttt‐3′
braS	5′‐gcggatccactagcacttggcgttatt‐3′	5′‐tcaagctttctcgcatacttaagtgca‐3′
vraD	5′‐cgcggatccttcgttgcgattatgggg‐3′	5′‐cgcaagcttaaacttgctgcaaccgga‐3′
Construction of the plasmid for gene complementation		
braRS‐pCL15	5′‐cgctgcagctatactttatatccgaca‐3′	5′‐aaggatccactagtatgcttacaatatt‐3′
braR‐pCL15	5′‐cgctgcagctatactttatatccgaca‐3′	5′‐aaggatcctgcattcaccctatacttta‐3′
braS‐pCL15	5′‐ttaaagcttagaaaaatgtcggatataaag‐3′	5′‐aaggatccactagtatgcttacaatatt‐3′
braXRS‐pCL8	5′‐cgcaagcttgtgacagaactaaaaaccg‐3′	5′‐aaggatccactagtatgcttacaatatt‐3′
Construction of the plasmid for reporter assay using β‐galactosidase		
braRS‐p	5′‐cggggatccgtgacagaactaaaaaccg‐3′	5′‐ttcagaaggcattttccacctcaaattatatt‐3′
vraDE‐p	5′‐acaggatccatcacttagaaagcacca‐3′	5′‐ttcagaaggcatagtctcactccttttgtat‐3′
vraDE‐p1	5′‐acaggatccatcacttagaaagcacca‐3′	5′‐aatgtttgaacctatcgctacgtagtag‐3′
vraDE‐p2	5′‐gtagcgataggttcaaacattgaattgtaa‐3′	5′‐ttcagaaggcatagtctcactccttttgtat‐3′
lacZ‐braRS‐p	5′‐ggagtgagactatgccttctgaacaatgg‐3′	5′‐ttggatccccacaactagaatgcagtg‐3′
lacZ‐vraDE‐p	5′‐ggagtgagactatgccttctgaacaatgg‐3′	5′‐ttggatccccacaactagaatgcagtg‐3′
Primers used for RACE	MW2546‐S1: 5′‐cttaaaaaatggaattacggt‐3′	MW2546‐A1: 5′‐acggctcttgatttgaactt‐3′
	MW2546‐S2: 5′‐ttgctagatattaatttgcc‐3′	MW2546‐A2: 5′‐cgctcctctaaaatagac‐3′
	MW2546‐5′ phosphate RT primer: 5′‐ccccatttgtattgc‐3′	
Amplification of DNA fragments used in gel shift assay		
vraDE‐F1	5′‐atcacttagaaagcacca‐3′	5′‐ccgtatgtttttgaaacat‐3′
vraDE‐F1′‐upper	5′‐atcacttagaaagcacca‐3′	5′‐aatgtttgaacctatcgctacgtagtag‐3′
vraDE‐F1′‐lower	5′‐gtagcgataggttcaaacattgaattgtaa‐3′	5′‐ccgtatgtttttgaaacat‐3′
Construction of the plasmid for recombinant protein		
rBraR	5′‐gcttatccatgaaaatattaattgttgaag‐3′	5′‐gcaagcttctatactttatatccgacat‐3′
rVraD	5′‐cgcggatccatgacaatattatcagtgc‐3′	5′‐cgcaagcttttaaatgtcatttgagacac‐3′
Primers for quantitative PCR		
*braR*	5′‐ttaaccaacatcaacctcag‐3′	5′‐ccccatttgtattgccat‐3′
*vraD*	5′‐cacttgccaaattccgta‐3′	5′‐aatacctaatgctgtcgtga‐3′
*gyrB*	5′‐aggtcttggagaaatgaatg‐3′	5′‐caaatgtttggtccggtt‐3′
Primers used for DNA sequence		
braRS‐seq‐F1	5′‐gtgacagaactaaaaaccg‐3′	—
braRS‐F561	5′‐aaaaaatggaattacggtg‐3′	—
braRS‐F1153	5′‐gatataaagtatagggtga‐3′	—
braRS‐F1730	5′‐aagtattaactgacgttag‐3′	—
braRS‐F2156	5′‐aaatgaagtgcatgcca‐3′	—
braRS‐seq‐R	—	5′‐atgtaattgtactgccaact‐3′
vraD‐seq‐FR	5′‐atcacttagaaagcacca‐3′	5′‐aatacctaatgctgtcgtga‐3′
MW2543‐seq‐F1	5′‐aagtattaactgacgttag‐3′	—
MW2543‐F551	5′‐tatttcaagagattcatcaa‐3′	—
MW2543‐F1101	5′‐attgattacgaatgattatg‐3′	—
MW2543‐F1703	5′‐ccagtcgttagtattgcc‐3′	—
MW2543‐F2111	5′‐acttgacgcacatgcg‐3′	—
MW2542‐seq‐R	—	5′‐ttgcgttgttgatgaataa‐3′

### 2.4 DNA sequences of the braRS, MW2543‐42 (braAB), and MW2620 (vraD) regions

Primers were designed to amplify the *braRS*, *braAB,* and *vraD* genes with their corresponding flanking regions, including promoter regions. In addition, primers were designed to amplify the *vraD* promoter region (Table 2). To prepare chromosomal DNA from the mutant strains, the cells from 1 ml of overnight cultures were collected. The cells were resuspended in 100 µl of 10 mM Tris‐HCl (pH 6.8) containing 10 µg lysostaphin (Sigma‐Aldrich) and incubated at 37°C for 20 min followed by an incubation at 95°C for 15 min. After centrifugation, the cell lysates were used as template DNA for PCR. PCR was performed using the Takara Ex Taq system, and the amplicons were purified using a QIAquick kit (Qiagen, Hilden, Germany). The nucleotide sequences of each DNA fragment were determined using specific primers, the sequences of which are listed in Table 2.

### 2.5 Inactivation of *braRS* in the mutant and its complementation

The method used to inactivate *braRS* with the thermosensitive plasmid pCL52.1 was described previously (Kawada‐Matsuo, Yoshida, et al., 2013). For gene complementation, the isopropyl‐β‐d‐thiogalactopyranoside (IPTG)‐inducible vector pCL15 was used. A DNA fragment for complementation was PCR‐amplified using chromosomal DNA from the wild‐type or mutant strains described above. The DNA fragment was cloned into pCL15 and transformed into *E. coli *XL‐II competent cells. The obtained plasmid was electroporated into *S. aureus* RN4220 and was subsequently transduced into the mutant strain using the phage 80α.

### 2.6 Analysis of the *vraDE* and *braXRS* promoter activities in nisin‐resistant mutants using a reporter system

Before the reporter assay, we identified the promoter region of *braXRS* using the rapid amplification of cDNA ends (RACE) method. RACE was performed using a 5′‐Full RACE Core Set (Takara Bio Inc., Ohtsu, Japan) according to the manufacturer's protocol, and the primers used in this assay are listed in Table 2. To analyze the *braXRS *and *vraDE *promoter activities, the respective promoter regions were fused to the β‐galactosidase gene using a PCR method. Briefly, the promoter regions and the β‐galactosidase gene were PCR‐amplified such that the downstream primer of the promoter region and the upstream primer of the β‐galactosidase gene contained ten overlapping nucleotides to allow the two PCR fragments to be joined together. After the first PCR, the two resulting PCR fragments were mixed and heated at 95°C, after which they were cooled to 37°C. Next, a second PCR was performed to amplify the fused fragment using primers listed in Table 2. The fragment was cloned into pLI50, a shuttle vector for *E. coli* and *S. aureus*, and the resulting plasmid was electroporated into *S. aureus* RN4220. Next, the plasmid was transduced into several *S. aureus *strains using the method described above. β‐Galactosidase assays were performed with a SensoLyte ONPG β‐Galactosidase Assay Kit (ANASPEC, CA, USA).

### 2.7 Expression of *braR* and *vraD*

Quantitative PCR and immunoblotting were performed to assess the expression of *braR*/BraR and *vraD*/VraD. The *S. aureus* strains were cultured overnight, and aliquots (containing 10^8^ cells) were inoculated into 5 ml of fresh TSB and then grown at 37°C with shaking. When the optical density reached 0.5 at 660 nm, nisin A (64 µg/ml) was added to the bacterial culture. After incubating for 15 min (for quantitative PCR) and 2 hr (for immunoblotting), the bacterial cells were collected. For quantitative PCR, RNA extraction, cDNA synthesis, and PCR were performed as described above. For immunoblotting, antiserum against VraD was obtained by immunizing mice with the recombinant protein as described previously (Kawada‐Matsuo, Oogai, et al., 2013). Briefly, histidine‐tagged recombinant VraD (rVraD) was constructed for the immunization. The DNA fragment encoding VraD was amplified with the specific primers listed in Table 2 and was subsequently cloned into pQE30 (Qiagen, Tokyo, Japan), with the resulting plasmid transformed into *E. coli* M15 (pREP4). The rVraD protein was purified according to the manufacturer's instructions. The bacterial cells were resuspended in 200 µl of Tris‐HCl (pH 6.8) containing 10 µg lysostaphin and were incubated for 20 min at 37°C and then at 95°C for 10 min. After centrifugation, the supernatant was mixed with equal volume of sample loading buffer and the proteins were resolved by 15% SDS‐polyacrylamide gel electrophoresis (PAGE). Next, the proteins were transferred to a nitrocellulose membrane. After blocking with 2% skim milk in Tris‐buffered saline (TBS; 20 mM Tris, 137 mM NaCl [pH 8.0]) containing 0.05% Tween 20 (TBS‐T), the membrane was incubated with specific antiserum (diluted 1:1,000 in 1% skim milk in TBS‐T) for 1 hr at 37°C. Next, membrane was washed with TBS‐T and incubated with horseradish peroxidase‐conjugated anti‐mouse IgG (diluted 1:1,000 in TBS‐T) (Promega, Madison, WI, USA) for 1 hr at 37°C. The membrane was then washed five times with TBS‐T, and the protein band reacting with the antiserum was detected using a chemiluminescence detection system (PerkinElmer, Waltham, MA, USA).

### 2.8 Electrophoretic mobility shift assay

For the electrophoretic mobility shift assay (EMSA), 6× histidine‐tagged recombinant BraR (rBraR) was utilized. A DNA fragment encoding BraR was amplified with the specific primers listed in Table 1 and was subsequently cloned into pQE30 (Qiagen). The plasmid was then transformed into *E. coli* M15 (pREP4), and the recombinant protein was purified according to the manufacturer's instructions. Purified protein was phosphorylated with a method described elsewhere (Gao, Gusa, Scott, & Churchward, 2005). The rBraR protein was incubated for 2 hr at room temperature in 50 mM Tris‐HCl (pH 8.0), 10 mM MgCl2, 3 mM dithiothreitol, and 32 mM acetyl phosphate. To assess the binding of rBraR to the region upstream of *vraDE*, an EMSA was performed as described previously (Mazda et al., 2012). A DNA fragment encompassing the region upstream of *vraDE* and a fragment lacking the binding region were amplified with the specific primers listed in Table 2. The DNA fragments were labeled at the 3′ end with digoxigenin (DIG) using a DIG Gel Shift Kit, 2nd Generation (Roche, Mannheim, Germany). The DIG‐labeled fragment (5 ng) was incubated with the MW2‐SAN8 (*S. aureus* nisin A‐resistant strain 8)‐rBraR protein (50 mM) in the labeling buffer provided with the kit. After native PAGE on a 6% polyacrylamide gel, the DNA fragments were transferred to a positively charged nylon membrane (Roche, Mannheim, Germany) and visualized according to the manufacturer's protocol.

## 3 RESULTS

### 3.1 Isolation of *S. aureus* strains with high levels of nisin A resistance and *VraD* expression

**Figure 1:**
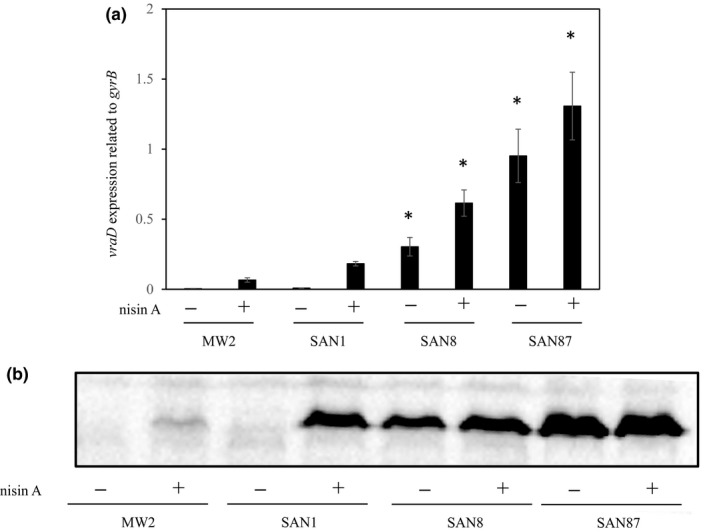
Expression of *vraD* in the MW2 and nisin A‐resistant strains. Protein and mRNA expression levels of *vraD* were evaluated by immunoblotting and quantitative PCR, as described in the Section 2. (a) Quantitative analysis of *vraD* expression in the MW2, SAN1, SAN8, and SAN87 strains. *p < 0.05, as determined by Dunnett's post hoc tests compared to untreated MW2. (b) Immunoblotting analysis of VraD expression in the MW2, SAN1, SAN8, and SAN87 strains

To obtain *S. aureus* MW2 mutants with high nisin A resistance, cells were exposed to increasing nisin A concentrations (1st, 256 µg/ml; 2nd, 1,024 µg/ml; and 3rd, 2,048 µg/ml). All 21 strains isolated from three independent experiments (experiments 1–3) showed a higher MIC of nisin A than the wild‐type strain. The spontaneous mutant strains exhibited MICs for nisin A from 1,024 to 8,192 µg/ml. Next, the expression of *vraD* was investigated by quantitative PCR. In experiment 1, one strain exhibited high levels of *vraD* expression in the presence of nisin A compared to the wild‐type strain, whereas the other six strains showed similar expression patterns as the wild‐type strain. In experiments 2 and 3, all 14 strains exhibited high levels of *vraD* expression compared to the wild‐type strain in the absence and presence of nisin A. We selected one strain from each experiment and designated them as SAN1, SAN8, and SAN87. Figure 1 shows the mRNA (a) and protein (b) expression of *vraD* in the MW2, SAN1, SAN8, and SAN87 strains. In the MW2 wild‐type strain, VraD expression was induced by nisin A. In contrast, SAN8 and SAN87 showed constitutively high VraD expression, even in the absence of nisin A, while SAN1 showed a higher VraD expression of in the presence of nisin A.

### 3.2 DNA sequence of the* braXRS*, *braAB*, and* vraD* regions

**Figure 2:**
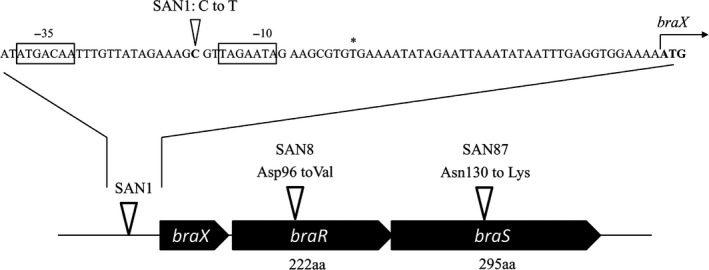
Mutation sites in the *braXRS *region in isolated mutants. The mutation sites in the *braXRS* region in the isolated mutants are indicated by white arrows. The nucleotide sequence upstream of *braXRS* in the MW2 strain is shown, with the −35 and −10 regions indicated in the box. The transcription initiation start sites are labeled with an asterisk, and the ATG translation initiation codons are indicated in bold

The DNA sequences of the *braRS*, *braAB,* and *vraD* regions in the SAN1, SAN8, and SAN87 strains were determined. In the SAN1 strain, only one mutation was observed in the promoter region of *braXRS* (Figure 2). In the SAN8 strain, one mutation in the *braR* region was observed that resulted in the replacement of aspartic acid at position 96 to valine (Figure 2). In the SAN87 strain, one mutation in the *braS* region was observed that resulted in the replacement of asparagine at position 130 to lysine (Figure 2). No mutations were detected in any of the mutants in the *vraD *promoter region or in *braAB*.

### 3.3 Susceptibility of strains to various antibacterial agents

We evaluated the MICs of various antibacterial agents against MW2, the nisin A‐resistant mutants, their *braRS*‐inactivated mutants, and the *braRS*‐complemented strains (Table 3). The inactivation of *braRS* in the SAN1, SAN8, and SAN87 strains caused a decrease in the MIC of nisin A to the same level as that observed in the *braRS*‐inactivated MW2 strain (MM2070). When the *braRS* genes of the individual SAN1, SAN8, and SAN87 were complemented in each mutant, the MICs of nisin A in each complemented strain were restored to nearly the same levels as those observed in the individual SAN1, SAN8, and SAN87 mutant strains. As for the bacitracin and gallidermin MICs, the SAN1, SAN8, and SAN87 strains showed a twofold increase in MIC compared to the wild‐type strain. In addition, the inactivation of *braRS* in the SAN1, SAN8, and SAN87 strains caused a decrease in the MIC of both antibiotics.

**Table 3:** Susceptibility of *Staphylococcus aureus* mutants to various antibacterial agents

Strain	Genotype	MIC (µg/ml)		
		Nisin A	Bacitracin	Gallidermin
MW2	Wild type	512	64	16
MM2070	*braRS *inactivation in MW2	128	32	8
MM2156	*braS *inactivation in MW2	128	32	8
MM2005	*vraD *inactivation in MW2	128	32	8
MM2139	*braRS* (MW2) complementation in MM2070	512	64	16
MM2141	*braRS* (SAN8) complementation in MM2070	4,096	64	16
SAN1	nisin A‐resistant mutant from MW2	1,024	64	16
MM2117	*braRS *inactivation in SAN1	128	32	8
MM2255	*braXRS* (MW2) complementation in MM2117	1,024	64	16
SAN8	nisin A‐resistant mutant from MW2	8,192	128	32
MM2120	*braRS *inactivation in SAN8	128	32	16
MM2147	*braRS* (SAN8) complementation in MM2120,	4,096	64	32
SAN87	nisin A‐resistant mutant from MW2	8,192	128	32
MM2256	*braRS *inactivation in SAN87	128	32	8
MM2258	*braRS* (SAN87) complementation in MM2256	4,096	64	32

Note

MIC: minimum inhibition concentration.

### 3.4 Expression of VraD in the mutants

**Figure 3:**
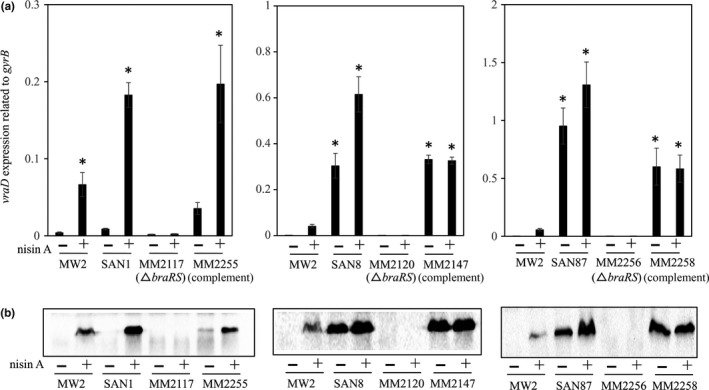
Expression of *vraD* in wild‐type MW2 and in the mutants. The protein and mRNA expression levels of *vraD* were evaluated by quantitative PCR (a) and immunoblotting (b), as described in the Section 2. The wild‐type strain MW2 and the SAN1, SAN8, and SAN87 mutants, as well as their *braRS*‐inactivated and *braRS*‐complemented strains, were investigated. *p < 0.05, as determined by Dunnett's post hoc tests compared to untreated MW2

We investigated the expression of VraD by immunoblotting and quantitative PCR (Figures 1 and 3). We observed similar protein and mRNA expression patterns in both experiments. The wild‐type MW2 strain showed inducible expression by nisin A. In contrast, VraD expression was very low in the SAN1 strain in the absence of nisin A, while VraD expression increased in the presence of nisin A, showing higher expression than that observed in the wild‐type strain (Figure 3). However, when *braRS* was inactivated in the SAN1 strain, VraD expression was not increased in the presence of nisin A, while in the *braRS*‐complemented strain, the VraD expression was similar with that observed in the SAN1 strain. In the SAN8 and SAN87 strains, VraD expression was higher in the absence and presence of nisin A than in the wild‐type strain with no nisin A added. When *braRS* was inactivated in the SAN8 and SAN87 strains, VraD expression in these strains was absent. In the complemented strains, VraD expression was recovered and showed similar expression levels as the SAN8 and SAN87 strains.

**Figure 4:**
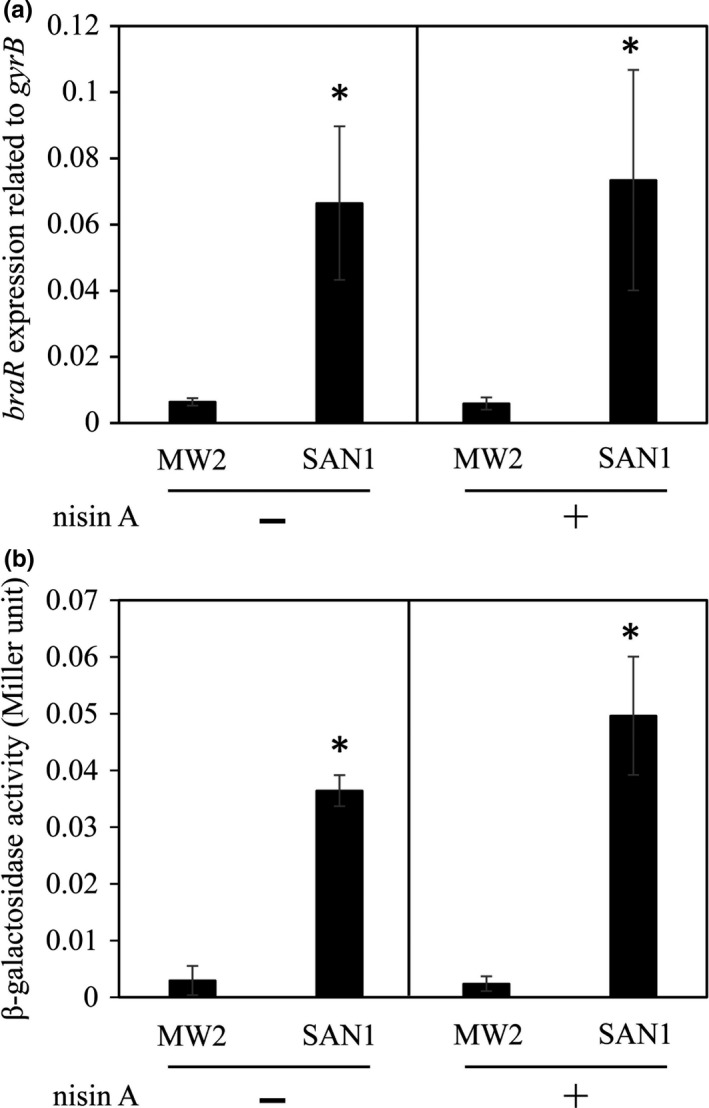
Expression of *braR* and the *braXRS* promoter activity in the MW2 and SAN1 strains. The expression of *braR* mRNA in the MW2 and SAN1 strains was evaluated by quantitative PCR (a) as described in the Section 2. The promoter activity of *braXRS *was evaluated using a β‐galactosidase reporter system (b), as described in the Section 2. *p < 0.05, as determined by *t* test

We next assessed the expression of *braR* in these strains and observed that only the SAN1 strain showed high *braR* expression compared to the wild‐type strain (Figure 4a), with the SAN8 and SAN87 strains showing similar expression as the wild‐type strain (data not shown).

### 3.5 *braXRS* and* vraDE* promoter activities

Since a point mutation in the SAN1 strain was observed in the *braXRS* promoter region, and the expression of *braR* was observed to be increased compared to that in the wild‐type strain by quantitative PCR (Figure 4a), we hypothesized that the *braXRS* promoter activity was increased in the SAN1 strain. We investigated the *braXRS* promoter activity in the wild‐type and SAN1 strains, and the activity was higher in the SAN1 strain than in the wild‐type strain (Figure 4b).

**Figure 5:**
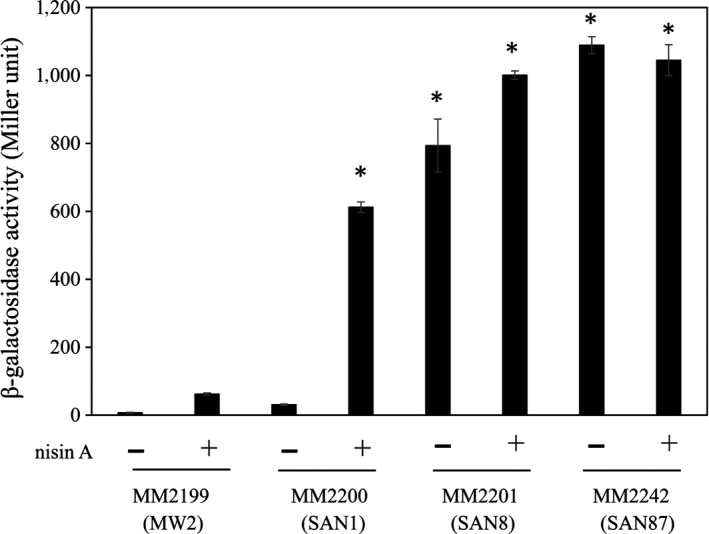
Activity of the *vraDE* promoter in the mutants. The *vraDE* promoter activity was evaluated using a β‐galactosidase reporter system as described in the Section 2. The plasmid for the reporter assay was constructed by fusing the *vraDE* promoter region with the gene encoding β‐galactosidase. Next, the plasmid was transduced into various strains, and β‐galactosidase activity was evaluated. *p < 0.05, as determined by Dunnett's post hoc tests compared to untreated MW2

We also investigated the *vraDE* promoter activity in the SAN1, SAN8, and SAN87 strains. The results were similar to those observed in the quantitative PCR and immunoblotting analyses. The SAN8 and SAN87 strains exhibited higher *vraDE* activity than the wild‐type strain in the absence and presence of nisin A, while the SAN1 strain showed higher activity only in the presence of nisin A (Figure 5).

### 3.6 Effect of the *BraR* mutation (in the SAN8 strain) on *vraD* expression and nisin A susceptibility

**Figure 6:**
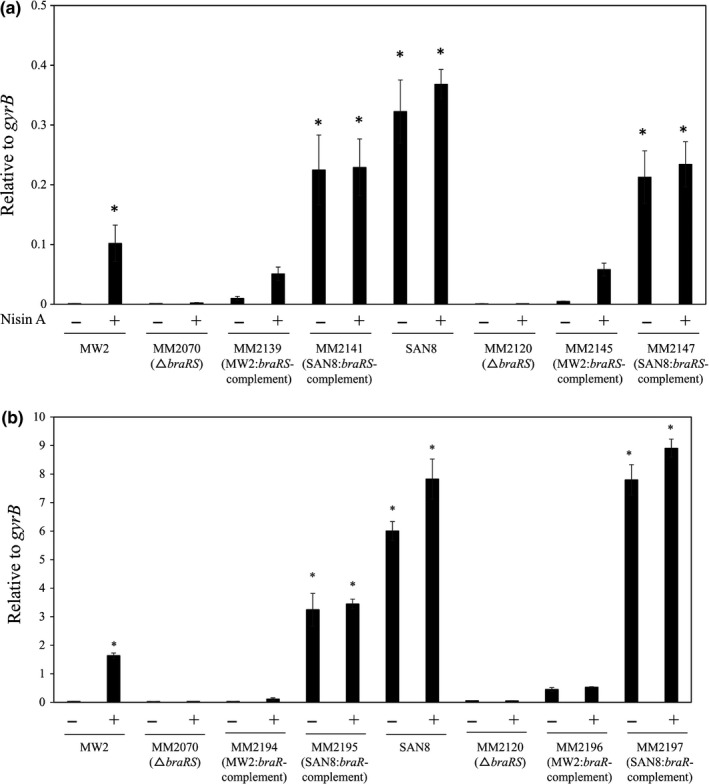
Effect of the mutated *braR* gene on the expression of *vraD*. The expression of *vraD* in the MW2, SAN8, and *braRS*‐inactivated strains and in the MW2 or SAN8 *braRS*‐inactivated strains complemented with *braRS* (MW2 or SAN8) (a) or *braR* (MW2 or SAN8) (b) was investigated by quantitative PCR as described in the Section 2. *p < 0.05, as determined by Dunnett's post hoc tests compared to untreated MW2

Using *braRS* from MW2 or SAN8, we complemented the *braRS*‐inactivated MW2 and SAN8 strains. We observed that complementation using *braRS* from the SAN8 (MM2147) strain but not MW2 (MM2145) resulted in almost identical *vraD* expression levels as was observed in the SAN8 strain (Figure 6a). In addition, when *braRS* from the SAN8 strain was introduced into the *braRS*‐inactivated MW2 strain (MM2141), *vraD* expression was significantly increased to almost identical levels as observed in the SAN8 strain (Figure 6a). Next, believing that only mutated BraR from the SAN8 strain affected the expression of VraD, we constructed strains harboring only the *braR* gene derived from the MW2 wild‐type or SAN8 strains in the *braRS*‐inactivated MW2 and SAN8 strains. In the strains possessing the *braR* gene from the SAN8 strain, an increase in *vraD* expression was observed in the *braRS*‐inactivated MW2 strain (MM2195), while increased *vraD* expression was not observed in the strain possessing the *braR* gene from the MW2 strain (MM2196) (Figure 6b). Furthermore, we assessed the MIC of nisin A in these strains and observed that the MM2195 and MM2197 mutants showed increased nisin A MICs, the same as that observed for the SAN8 strain, while for the MM2194 and MM2196 strains, the introduction of *braR* from the MW2 wild‐type strain did not increase the MIC of nisin A (Table 4). We also transduced *braR* from the SAN8 strain into RN4220, a methicillin‐susceptible strain. An RN4220 strain harboring *braR *from the SAN8 strain showed an increase in the MIC of nisin A, whereas an RN4220 strain harboring *braR* from the MW2 strain showed no alteration in the MIC (Table 4).

**Table 4:** Effect of mutated BraR of SAN8 on the susceptibility to nisin A

Strain	Genotype	MIC (µg/ml)
		Nisin A
MW2	Wild type	512
MM2070	*braRS *inactivation in MW2	128
MM2194	*braR* (MW2) in MM2070	512
MM2195	*braR* (SAN8) in MM2070	4,096
MM2196	*braR* (MW2) in MM2120	512
MM2197	*braR* (SAN8) in MM2120	4,096
RN4220	Wild‐type, laboratory strain	512
MM2186	*braR* (MW2) in RN4220	512
MM2187	*braR* (SAN8) in RN4220	4,096

Note

MIC: minimum inhibition concentration.

### 3.7 Binding of the wild‐type and mutated BraR proteins to the upstream region of* vraDE*

**Figure 7:**
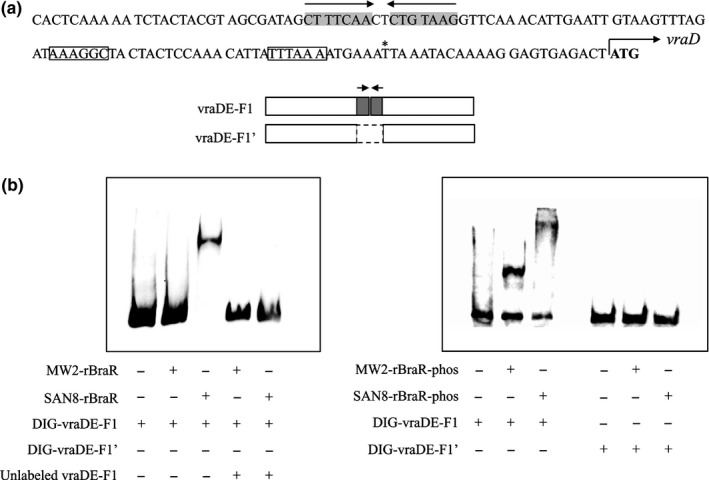
BraR electrophoretic mobility shift assay (EMSA). (a) The nucleotide sequence of the *vraDE* promoter region and the DNA fragments used in this study. DNA fragments with or without the BraR‐binding site were used. Gray shadow, palindromic sequence: squares, −35, −10 box; *, *vraD* transcriptional start site; bold, *vraD* translation initiation codon. (b) EMSA of BraR using two DNA fragments. Fragments were labeled with DIG and incubated with recombinant unphosphorylated (rBraR, left) or phosphorylated BraR protein (rBraR‐phos, right) as described in the Section 2. After electrophoresis, DNA bands were detected as described in the Section 2

**Figure 8:**
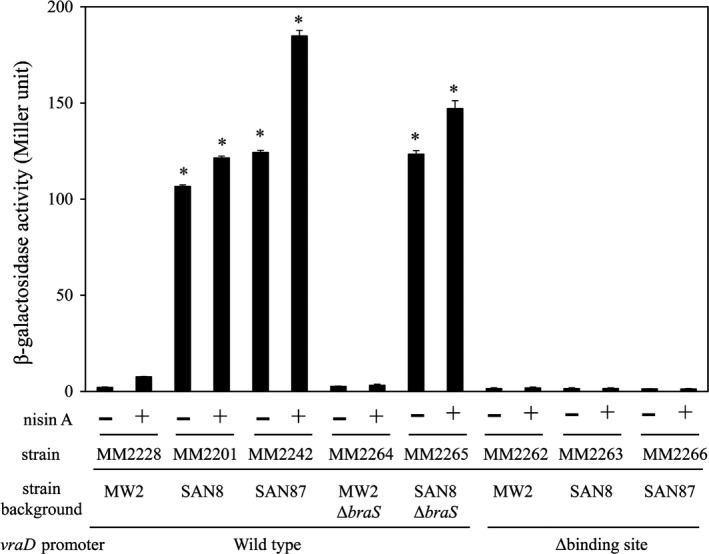
The *vraDE *promoter activity in the *braS*‐inactivated mutants. The *vraDE* promoter activity was evaluated using a β‐galactosidase reporter system as described in the Section 2. The plasmid for the reporter assay was constructed by fusing the wild‐type *vraD* promoter region or the *vraD* promoter region with the BraR‐binding site deleted with the gene encoding β‐galactosidase. Next, the plasmid was transduced into various strains, and β‐galactosidase activity was evaluated. *p < 0.05, as determined by Dunnett's post hoc tests compared to untreated MW2

Electrophoretic mobility shift assay showed the binding of phosphorylated MW2‐rBraR and SAN8‐rBraR (phosphorylated and nonphosphorylated) with the upstream region of *vraDE*. Figure 7a shows the *vraDE* region to which BraR bound and the fragments utilized with (vraDE‐F1) or without (vraDE‐F1′) the BraR‐binding region. Compared to MW2‐BraR, SAN8‐BraR had a strong affinity for the DNA‐binding region upstream of *vraDE* (Figure 7b). The addition of excess unlabeled *vraDE* fragments caused the loss of the band containing SAN8‐rBraR bound to the Dig‐labeled *vraDE* fragment (Figure 7b, Left). When the BraR‐binding region in the region upstream of *vraDE* was deleted, both BraR proteins could not bind to the fragment (Figure 7b, Right). In the reporter assay, the results were similar to the EMSA assay results (Figure 8). In the *braS*‐inactivated SAN8 mutant, the promoter activity of *vraD* exhibited high expression, showing a similar level of activity as the SAN8 strain, while the promoter activity was not increased by the addition of nisin A in the *braS*‐inactivated MW2 strain. The deletion of the BraR‐binding site in the *vraD* promoter region in the SAN8 and SAN87 strains resulted in the loss of promoter activity in the absence and presence of nisin A.

## 4 DISCUSSION

In this study, we isolated three spontaneous mutants (SAN1, SAN8, and SAN87) exhibiting high levels of nisin A resistance and VraDE expression, all of which possessed single mutations in the *braXRS* region. Gene inactivation and complementation experiments clearly demonstrated that the point mutation in *braXRS* was directly associated with the high resistance of the mutants to nisin A. Two mutants, SAN8 and SAN87, showed constitutively high VraDE expression, even in the absence of nisin A. In contrast, the SAN1 strain showed low VraDE expression in the absence of nisin A but higher expression in the presence of nisin A than the wild‐type strain. In previous reports, ApsRS, a TCS in *S. aureus*, was also associated with nisin A susceptibility (Kawada‐Matsuo, Yoshida, et al., 2013). ApsRS regulates the expression of *dlt *and *mprF* to suppress the negative charge of the bacterial cell surface (Meehl, Herbert, Götz, & Cheung, 2007; Sakoulas et al., 2002). However, mutations in *apsRS* were not detected in the mutants isolated in this study.

In the SAN1 strain, only one point mutation was observed between the −35 and −10 box in the *braXRS* promoter region. We observed an increase in *braRS* expression in the SAN1 strain compared to that detected in the wild‐type strain (Figure 4a). The reporter assay also revealed that the *braXRS* promoter activity in the SAN1 strain was 10 times higher than that observed in the wild‐type strain (Figure 4b). Based on these results, we speculated that high amount of BraRS in the SAN1 strain increased the level of phosphorylated BraR by the addition of nisin A, which resulted in a higher induction of VraDE in response to nisin A than in the wild‐type strain, although we did not quantify the level of phosphorylated BraR.

In the SAN8 strain, a BraR mutation at amino acid position 96 (aspartic acid to valine) (BraR^M^) caused the expression of VraDE to be constitutively increased. When the BraR^M^ allele was introduced into the *braRS*‐inactivated MW2 strain (MM2195), this strain showed a similar MIC for nisin A to that observed for the SAN8 strain (Table 3). In addition, inactivation of *braS* alone in the SAN8 strain (MM2116) did not decrease the MIC of nisin A (data not shown). Based on these results, we considered that unphosphorylated BraR^M^ could enhance the expression of VraDE. In the reporter assay, the *vraDE* promoter deleted of the BraR‐binding region (MM2263) had no activity (Figure 8). These results suggested that BraR^M ^bound to the same binding region upstream of *vraDE* as the native BraR. The EMSA assay also showed the binding affinity of BraR^M^ to the upstream *vraDE* region and that its affinity was higher than that of the native BraR.

**Figure 9:**
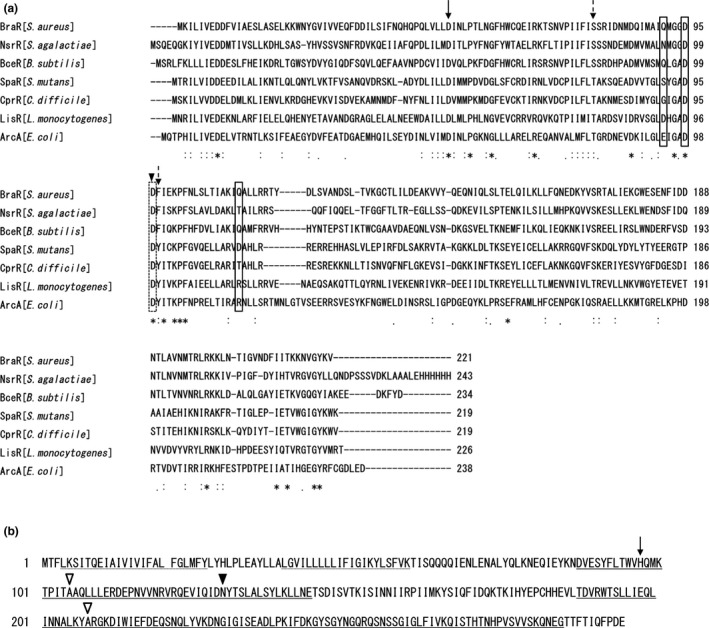
Protein alignments of BraR with other proteins and amino acid sequence of BraS. Protein alignment of BraR with other response regulators exhibiting homology with BraR (a). The active site aspartate residue (arrow), the two switch residues (dashed arrows), and the dimer interface regions (shown in the box and the dashed box) are shown. The triangle represents the mutation site in the mutant. Protein sequence of BraS (b). The dashed underline, double underline, and underline represent the region for the membrane‐spanning region, histidine kinase domain, and ATPase domain region, respectively. The active site histidine residue (the arrow), the mutation site in the SAN87 strain (black triangle), and the mutation sites reported previously (white triangle) are shown

Khosa, Hoeppner, Gohlke, Schmitt, and Smits (2016) reported the structure of NsrR from *Streptococcus agalactiae*, which showed homology with BraR from *S. aureus*. NsrRK is a TCS responsible for the expression of *nsr* and *nsrFP*, which are involved in nisin resistance (Khosa, AlKhatib, & Smits, 2013). Figure 9a shows an amino acid sequence alignment of response regulators reported to be associated with lantibiotic resistance, including BceR from *Bacillus subtilis* (Staroń, Finkeisen, & Mascher, 2011), SpaR/LcrR from *S. mutans* (Kawada‐Matsuo, Oogai, et al., 2013), CprR from *Clostridium difficile *(McBride & Sonenshein, 2011; Suárez, Edwards, & McBride, 2013), and LisR from *Listeria monocytogenes* (Cotter, Emerson, Gahan, & Hill, 1999). According to the structural analysis of NsrR (Khosa et al., 2013), we showed the active site aspartate residue, the two switch residues, and the dimer interface regions of *S. aureus* BraR and other response regulators that were shown to be associated with nisin A susceptibility (Figure 9a). The BraR^M^ mutation site at position 96 is an aspartic acid residue (black triangle) that is adjacent to a phenylalanine residue, which is a conserved amino acid residue involved in a switch residue (dashed arrow). In a structural analysis of NsrR and ArcA, the phosphorylation of an aspartic acid causes a conformational change in two amino acid residues called switch residues (shown in the box in Figure 9a; Khosa et al., 2013; Toro‐Roman, Mack, & Stock, 2005). This conformational change induces the response regulator to form a dimer. In addition, four amino acid residues (three boxes and a dashed box in Figure 9a), including the aspartic acid at position 96 (dashed box), are important for stabilising the dimer formation by forming salt bridges. Due to the different properties of aspartic acid (hydrophilic and acidic) and valine (hydrophilic and nonpolar), the amino acid replacement at position 96 (Asp to Val) in BraR causes a conformational change, especially at the dimer interface region. This structural change is presumed to cause BraR to form a dimer in the absence of phosphorylation, causing the unphosphorylated BraR^M^ to bind upstream of *vraDE*.

In the SAN87 strain, the BraS mutation at position 130 (asparagine to lysine) (BraS^M^) caused VraDE to be expressed constitutively. Since the *braR* gene in strain SAN87 had no mutations, we believe that BraR is phosphorylated even in the absence of nisin A in the SAN87 strain. Figure 9b shows an amino acid sequence alignment of sensor proteins that were reported to be associated with nisin resistance. A sensor protein consists of three regions, a sensing region, which includes the transmembrane region, a histidine kinase region, and an ATPase region. Conformational changes in the sensor region by environmental stimuli cause the catalytic centers of the sensor kinase to become activated and generate phosphorylated BraS. Next, a phosphorylation relay occurs where phosphorylated BraS phosphorylates BraR to generate phosphorylated BraR. The mutation site in BraS^M ^(black triangle) is in the histidine kinase region next to the sensor region. This mutation may affect the conformation of the BraS histidine kinase region, causing activation of the catalytic region without the need for nisin A stimulation. Previously, Blake KL reported the isolation of a nisin‐resistant *S. aureus* strain and identified a mutation in *braS* (Blake, Randall, & O'Neill, 2011). They identified two mutation sites at positions 105 (A to T) and 208 (A to E), indicated as white triangles. Positions 105 and 208 AA are within the histidine kinase region and ATPase region, respectively. Although they did not investigate the expression of VraDE, the mutation of BraS may allow a conformational change to occur that mimics the phosphorylated BraS protein without the need for nisin A stimulation.

In conclusion, we obtained three spontaneous *S. aureus* MW2 mutants with high levels of nisin A resistance by exposing cells to a sub‐MIC of nisin A. Interestingly, the mutants harbored single point mutations in the *braRS* region that induced constitutive expression of the target gene without the need for environmental stimuli. Our findings also provide new insights into the key amino acids of BraRS required for nisin A resistance in *S. aureus*.

## CONFLICT OF INTEREST

The authors declare that they have no competing interests.

## AUTHORS CONTRIBUTION

KA, MK‐M, KN, and HK designed the research and analyzed data. KA, MK‐M, YO, and HK performed the experiments. KA, MK‐M, and HK wrote the manuscript. HK directed the research.

## ETHICS STATEMENT

None required.

## Data Availability

All data are provided in full in the results section of this paper.
